# Revocable and Traceable Decentralized ABE for P2P Networks

**DOI:** 10.3390/e28010077

**Published:** 2026-01-09

**Authors:** Dan Gao, Huanhuan Xu, Shuqu Qian

**Affiliations:** 1 Department of Information Engineering, Anshun Technical College, Anshun 561000, China; 2 College of Computer Science and Technology, Guizhou University, Guiyang 550025, China; huanh_xu@163.com; 3 School of Mathematics and Computer Science, Anshun University, Anshun 561099, China; shuquqian@163.com

**Keywords:** attribute-based encryption, P2P networks, revocation, traceability

## Abstract

Ciphertext-Policy Attribute-Based Encryption (CP-ABE) technology provides fine-grained access control capabilities for P2P networks. However, its long-term development has been constrained by three major challenges: the trade-off between computational efficiency and functional completeness, decentralized trust security issues, and the problems of attribute revocation and traceability. This paper proposes a decentralized CP-ABE scheme based on multiple authorities (R-T-D-ABE). By leveraging three core techniques, including threshold distributed key generation, versioned attribute revocation, and identity-key binding verification, the scheme efficiently achieves both revocation and accountability while ensuring resistance against collusion attacks and forward/backward security. Security analysis demonstrates that the proposed scheme satisfies IND-CPA security under the Generic Group Model (GGM). Experimental results indicate that it not only guarantees efficient decentralized encryption and decryption but also realizes the dual functions of revocation and accountability, thereby providing a functionally complete and efficient access control solution for P2P networks.

## 1. Introduction

Peer-to-peer (P2P) networks, leveraging their decentralized nature, have demonstrated significant advantages in a wide range of applications, from blockchain systems [1,2,3] to critical infrastructure domains such as smart grids, distributed energy trading, and vehicular networks [4,5,6]. However, they also introduce new security challenges for access control [7,8]. Traditional access control techniques, which employ static permission assignment models [9], struggle to meet the demand for dynamic permission allocation in P2P networks [7,10]. To address this, researchers have introduced Attribute-Based Encryption (ABE), a concept first proposed by Sahai and Waters in 2005 [11]. Building upon this, Bethencourt et al. proposed Ciphertext-Policy Attribute-Based Encryption (CP-ABE) in 2007 [12]. This scheme allows data owners to directly specify access policies during encryption, enabling more flexible and fine-grained access control, and has been widely adopted to protect shared data security in P2P networks [13,14,15]. Despite its advantages, CP-ABE still faces three interrelated core challenges in the decentralized P2P environment, making it difficult for existing schemes to simultaneously achieve attribute revocation and user traceability under decentralization constraints without incurring efficiency bottlenecks.

First, there exists a difficult-to-reconcile contradiction between computational efficiency and functional completeness in ABE scheme design. Attribute matching in CP-ABE relies on bilinear pairing operations. Most current CP-ABE frameworks, including those based on classic schemes such as BSW CP-ABE [16], FAME CP-ABE [17], and ABGW CP-ABE [18], struggle to fundamentally overcome the efficiency bottleneck of large-scale attribute matching. On one hand, pursuing extreme efficiency often comes at the cost of functionality. For instance, optimizing data structures to improve efficiency may sacrifice policy flexibility [19], or relying on online/offline techniques may introduce centralized components [20]. Notably, even the fastest scheme like FABEO [21] does not consider implementing attribute revocation under a multi-authority setting. On the other hand, complex mechanisms introduced to enhance functionality, such as proxy re-encryption [22] or composite-order bilinear groups [23], significantly increase computational overhead, rendering many schemes impractical for deployment in P2P networks.

Second, the decentralized nature of P2P networks conflicts with the traditional centralized trust model of CP-ABE [24]. Multi-Authority CP-ABE (MA-CP-ABE) aims to address this by allowing multiple independent authorities to manage different attributes, thereby mitigating single points of failure [25,26,27,28]. However, existing approaches still face issues: some fail to completely eliminate centralized architecture [26], while others incur high key generation latency or additional computational and trust overhead due to the use of smart contracts [25], proxy nodes [28], or ring signatures [27].

Finally, the attribute revocation and user traceability functions essential for dynamic member management face a contradiction between privacy and efficiency in P2P networks. Efficient attribute revocation requires effective key and ciphertext update mechanisms to prevent replay attacks, but existing techniques are often limited due to coarse-grained revocation [29], reliance on trusted third parties [30], the need for additional delegated nodes [22], or dependency on specific communication protocols [31]. Simultaneously, user traceability is crucial for deterring attacks, yet existing schemes either suffer from high computational complexity when protecting privacy (e.g., requiring linear search [1]) or compromise decentralization for the sake of efficiency (e.g., relying on blockchain for permission table updates [32]), making it challenging to achieve both [33,34].

To address the aforementioned challenges, this paper proposes a Revocable and Traceable Decentralized ABE scheme (R-T-D-ABE) suitable for P2P networks. The core contribution of this work lies in its successful resolution of the efficiency bottleneck that arises from the simultaneous implementation of attribute revocation and user traceability within the highly dynamic and decentralized context of P2P networks. The main contributions are as follows:

- Design of an efficient decentralized key management mechanism for P2P networks. Utilizing distributed authority signatures and Shamir’s secret sharing technology, we achieve key generation and distribution without central coordination, eliminating single points of failure and enabling efficient encryption and decryption.
- Realization of real-time lightweight attribute revocation. Through version number control and coordination among distributed authorities, lightweight dynamic key updates within the P2P network are ensured, achieving attribute-level fine-grained real-time revocation.
- Proposal of a privacy-preserving and non-repudiable user traceability scheme. By binding user identities to their keys, our scheme enables fast and accurate tracing of key leakage sources without exposing user identities, effectively resolving the conflict between privacy and traceability efficiency.
- Provision of provable security guarantees. Through security proofs, our scheme is demonstrated to achieve IND-CPA security, collusion resistance, forward security, and backward security under the Generic Group Model (GGM).

The remainder of this paper is organized as follows. Section 2 reviews the preliminary knowledge. Section 3 elaborates on the detailed construction of the proposed R-T-D-ABE scheme. Security analysis and proofs are presented in Section 4. Section 5 discusses both theoretical and experimental evaluations. Finally, conclusions and future work are outlined in Section 6.

## 2. Preliminaries

### 2.1. Bilinear Maps

Let λ be the security parameter, and $G_{1}$, $G_{2}$, and $G_{T}$ be three multiplicative cyclic groups of prime order *p*. Let $g_{1}$ and $g_{2}$ be generators of $G_{1}$ and $G_{2}$, respectively. A bilinear map $e:G_{1}\times G_{2}\to G_{T}$ is a function satisfying:

- Bilinearity: $\forall u\in G_{1}$, $\forall v\in G_{2}$, $\forall a,b\in Z_{p}$, we have $e\left(u^{a},v^{b}\right)=e{\left(u,v\right)}^{ab}$.
- Non-degeneracy: $e\left(g_{1},g_{2}\right)\neq 1_{G_{T}}$.
- Computability: There exists an efficient algorithm to compute e(u,v) within deterministic polynomial time with respect to the security parameter λ.

### 2.2. Generic Group Model (GGM)

The Generic Group Model (GGM) [35,36] treats group elements as opaque handles, allowing adversaries to perform group operations only via oracles. Boneh et al. [37] extended it to bilinear groups. In our proof, we analyze security in an extended GGM that incorporates:

- Integration of Random Oracle: The hash function is modeled as a random oracle *H*.
- Embedding of Scheme-Specific Oracles: The adversary is allowed to access oracles $O_{\mathrm{mpk}}$, $O_{\mathrm{ct}}$, and $O_{\mathrm{sk}}$.
- Extended Adversarial Capabilities: Besides basic group operations, the adversary can also compute pairings through an oracle.

These extensions preserve the core limitation of GGM—that adversaries cannot directly manipulate the algebraic representation of group elements—while enabling the model to accurately reflect the security environment of the actual scheme. In Section 4.5, we prove that the scheme achieves IND-CPA security under the extended GGM by constructing a sequence of indistinguishable games ( ${\mathrm{Game}}_{0}$, ${\mathrm{Game}}_{1}$, ${\mathrm{Game}}_{2}$ ), with an advantage upper bounded by $O(Q^{2})/p$, where *Q* denotes the number of adversary queries and *p* is the group order. The proof also demonstrates that the scheme satisfies forward security, backward security, and collusion resistance.

### 2.3. Monotone Span Programs (MSP)

Let $Arr=\left\{{Arr}_{1},{Arr}_{2},\ldots ,{Arr}_{n}\right\}$ be an attribute set, and *A* be a monotone access structure on Arr, meaning *A* is a collection of non-empty subsets of Arr with the monotonicity property: if an authorized attribute set S∈A, then any superset $S^{\prime }⊃S$ is also authorized.

In this CP-ABE scheme, the access structure *A* is described by MSP, defined as follows:

- Matrix Representation: Let $M\in Z_{p}^{m\times n}$ be a matrix over the finite field $Z_{p}$, where *m* is the number of rows and *n* is the number of columns. The row labeling function ρ:[m]→Arr associates the *i*-th row of matrix *M* with an attribute in Arr, i.e., $\rho \left(i\right)={Arr}_{i}$.
- Authorization Set Determination: For a user’s attribute set *S*, let $I_{S}=\{i\in \left[m\right]:$ρ(i)∈S} denote the set of row indices whose associated attributes belong to *S*. Let $M_{S}$ be the submatrix of *M* consisting of all rows where $i\in I_{S}$. Given a target vector $v=\left(1,0,\ldots ,0\right)\in Z_{p}^{1\times n}$, if *S* is an authorized set (S∈A), there exists a weight vector $w\in Z_{p}^{1\times |S|}$ such that w·M=v holds; otherwise, *S* is unauthorized (S∉A).

### 2.4. FABEO

In 2022, Riepel and Wee proposed a fast attribute-based encryption (ABE) scheme achieving optimal adaptive IND-CPA security, based on asymmetric (Type-III) bilinear groups. The core idea is to allocate most computations to the group $G_{1}$ and to optimize the number of bilinear pairing operations during decryption. A brief description of the CP-ABE scheme in FABEO is given below.

- Setup: The system master key is $\alpha \overset{R}{\leftarrow }Z_{p}$. Define a hash function $H:U\cup \left\{0\right\}\to G_{1}$. Let $H\left(u\right)=g_{1}^{b[u]}$ for each attribute u∈U, and $h=H\left(0\right)=g_{1}^{b^{\prime }}$. The master public key is $\mathrm{mpk}=(p,G_{1},G_{2},G_{T},e,g_{1},g_{2},H,e{\left(g_{1},g_{2}\right)}^{\alpha }).$
- KeyGen: For an attribute set S⊆U, choose a random $r\overset{R}{\leftarrow }Z_{p}$ and generate the secret key $\mathrm{sk}=\left({\mathrm{sk}}_{1}=g_{1}^{\alpha }\cdot h^{r},\;{\mathrm{sk}}_{2}={\left\{H{\left(u\right)}^{r}\right\}}_{u\in S},\;{\mathrm{sk}}_{3}=g_{2}^{r}\right).$
- Encrypt: To encrypt a message *M* under an access structure $\left(M\in Z_{p}^{m\times n},\rho :\left[m\right]\to U\right)$, where τ denotes the maximum reuse count of attributes in M, choose random vectors $s=\left(s_{1},v\right)\overset{R}{\leftarrow }Z_{p}^{n}$ and $s^{\prime }\overset{R}{\leftarrow }Z_{p}^{\tau }$. The ciphertext is constructed as: ${\mathrm{ct}}_{0}=M\cdot e{\left(g_{1},g_{2}\right)}^{\alpha s_{1}},$ ${\mathrm{ct}}_{1}=g_{2}^{s_{1}},$ ${\mathrm{ct}}_{2,j}=g_{2}^{s^{\prime }\left[j\right]},$ ${\mathrm{ct}}_{3,i}=h^{M_{i}{\left(s_{1}∥v\right)}^{⊤}}\cdot H{\left(\rho (i)\right)}^{s^{\prime }\left[\rho \left(i\right)\right]},$ and $\mathrm{ct}=\left({\mathrm{ct}}_{0},\;{\mathrm{ct}}_{1},\;{\left\{{\mathrm{ct}}_{2,j}\right\}}_{j\in [\tau ]},\;{\left\{{\mathrm{ct}}_{3,i}\right\}}_{i\in [n_{1}]}\right).$
- Decrypt: If *S* satisfies (M,ρ), there exists a set of constants ${\left\{\gamma_{i}\right\}}_{i\in I}$ such that $\sum_{i\in I}\gamma_{i}M_{i}=\left(1,0,\ldots ,0\right)$. Decryption is performed as follows:
  $$e\left({\mathrm{sk}}_{1},{\mathrm{ct}}_{1}\right)\cdot \frac{\prod_{j\in [\tau ]}e\left(\prod_{\begin{array}{c} i\in I\\ \rho (i)=j \end{array}}{\left({\mathrm{sk}}_{2,\pi (i)}\right)}^{\gamma_{i}},\;{\mathrm{ct}}_{2,j}\right)}{e\left(\prod_{i\in I}{\left({\mathrm{ct}}_{3,i}\right)}^{\gamma_{i}},\;{\mathrm{sk}}_{3}\right)}=e{\left(g_{1},g_{2}\right)}^{\alpha s_{1}}.$$

FABEO incorporates the above CP-ABE scheme into the Pair Encoding Scheme ABE (PES-ABE) proof framework and proves that [21]: any PES-ABE scheme satisfying (1,1)-symbolic security automatically satisfies strong symbolic security. Consequently, under the Generic Group Model (GGM) and the Random Oracle Model (ROM), the scheme achieves optimal adaptive multi-ciphertext IND-CPA security.

### 2.5. PES-ABE

The Pair Encoding Scheme for Attribute-Based Encryption (PES-ABE) is a framework that modularizes security proofs [38,39], primarily comprising the following deterministic algorithms:

- ${\mathrm{Setup}}_{0}\left(\lambda ,X,Y\right)\to n$: On input the security parameter λ, the policy space X, and the attribute space Y, this algorithm outputs n∈N, specifying the number of hash attributes in the master secret key, which serves as a global public parameter.
- ${\mathrm{KeyGen}}_{0}\left(y\right)\to \left(k^{1},k^{2}\right)$: Given a user’s attribute set *y*, it outputs two linear functions $k^{1}:Z_{p}^{1+m+mn}\to Z_{p}^{m_{1}}$ and $k^{2}:Z_{p}^{m}\to Z_{p}^{m_{2}}$, where *m* is the length of the key’s random vector, $m_{1}$ denotes the number of $G_{1}$ elements in the key, and $m_{2}$ denotes the number of $G_{2}$ elements.
- ${\mathrm{Enc}}_{0}\left(x\right)\to \left(c^{1},c^{2}\right)$: Given an access structure *x* (specifically modeled as a Monotone Span Program (M,π) in this work), it outputs two linear functions $c^{1}:Z_{p}^{wn}\to Z_{p}^{w_{1}}$ and $c^{2}:Z_{p}^{w}\to Z_{p}^{w_{2}}$, where *w* is the length of the ciphertext’s random vector, $w_{1}$ is the number of $G_{1}$ elements in the ciphertext, and $w_{2}$ is the number of $G_{2}$ elements.

In a concrete ABE scheme, these deterministic algorithms are instantiated over bilinear groups, with computations performed in the exponent to generate the corresponding ciphertexts and keys.

It has been explicitly defined and proven within the FABEO scheme that any scheme satisfying symbolic security under the PES-ABE framework also satisfies *strong* symbolic security. Consequently, such a scheme achieves adaptive, multi-challenge IND-CPA security under both the Generic Group Model (GGM) and the Random Oracle Model (ROM).

Therefore, in our security proof, we first abstract the proposed R-T-D-ABE scheme into the PES-ABE framework and prove that it satisfies symbolic security. Based on the conclusion from FABEO, this is equivalent to satisfying strong symbolic security. Finally, leveraging this strong symbolic security, we prove that our scheme achieves IND-CPA security, collusion resistance, forward security, and backward security in the GGM.

## 3. R-T-D-ABE

### 3.1. System Mode

This scheme addresses the requirements for privacy preservation in peer-to-peer (P2P) networks. The proposed R-T-D-ABE scheme aims to achieve fine-grained access control, decentralized trust, dynamic revocation, and leakage traceability. Security is proven in the Generic Group Model (GGM) with the hash function modeled as a Random Oracle (ROM), achieving adaptive IND-CPA security with an optimal security bound.

**Figure 1:**
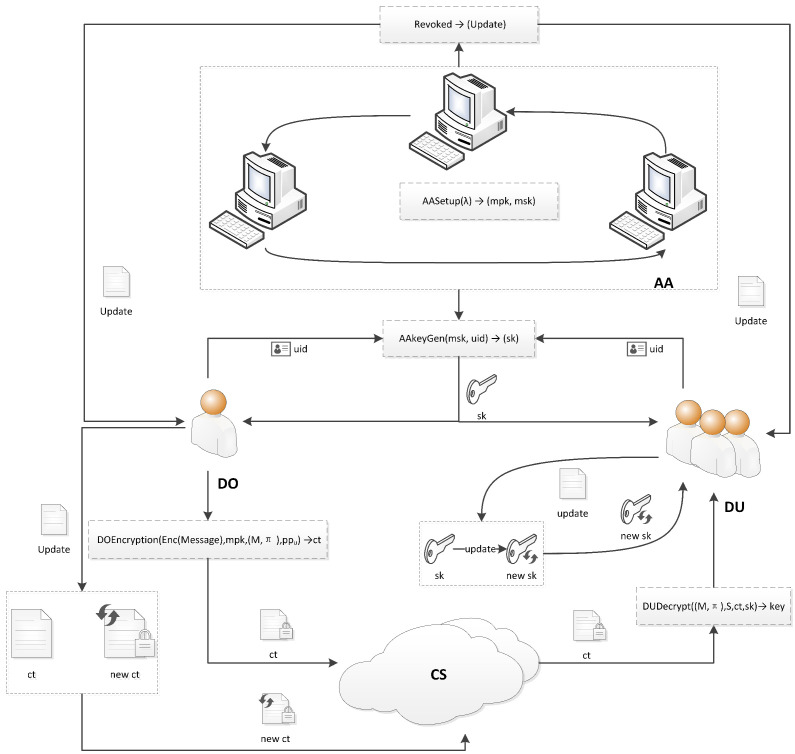
System framework diagram.

As illustrated in Figure 1, the proposed architecture comprises four core entities:

- Data Owners (DOs): Entities that encrypt sensitive data and define the access policies.
- Data Users (DUs): Entities that request and access data, with their permissions governed by their attributes.
- Authorization Authority Cluster (AA): A decentralized set of authorities that collectively manage user attributes and are responsible for key generation and updates.
- Cloud Server (CS): A service provider that offers storage and computational resources, hosting the encrypted data.

Its key operational phases detailed as follows:

Distributed Key Generation: Multiple Authorization Authorities (AAs) collaboratively generate the system master key and user private keys using distributed authority signatures and Shamir’s Secret Sharing technique. This process eliminates single points of failure and establishes a foundation for a decentralized trust framework.

Data Encryption and Upload: Data Owners (DOs) define access policies and encrypt sensitive data accordingly, then upload the resulting ciphertext to the Cloud Server (CS) for storage.

Data Download and Decryption: Data Users (DUs) can successfully decrypt and access the encrypted data if and only if their attribute set and key version number satisfy the access policy and version requirements embedded within the ciphertext.

Dynamic Attribute Revocation:

- Upon receiving a revocation request, the Authorization Authority cluster (AA) cooperatively generates key update information.
- Non-revoked users can subsequently use this information to independently update their credentials without any system downtime.

Leakage Traceability: The scheme incorporates unique identity markers into the cryptographic keys, enabling the multiple AAs to precisely trace the source of any private key leakage, thereby providing non-repudiation support for auditing purposes.

In summary, the proposed scheme exhibits three salient features:

- The elimination of single points of failure through a fully decentralized architecture;
- Support for dynamic, attribute-level privilege management;
- Built-in, efficient leakage traceability that enhances system accountability.

### 3.2. Scheme Construction

The scheme operates over public parameters $pp=(p,G_{1},G_{2},G_{T},e,g_{1},g_{2},H,\theta )$, where *p* is a large prime, $G_{1},G_{2},G_{T}$ are cyclic groups of order *p* with bilinear map $e:G_{1}\times G_{2}\to G_{T}$, $g_{1},g_{2}$ are generators, $H:\left[|\theta |+1\right]\to G_{1}$ is a hash function modeled as a random oracle (ROM), and θ is the attribute universe.

It provides six core algorithms:

- AASetup(λ)→(mpk,msk): Distributed system initialization.
- AAkeyGen(msk,uid,S)→sk: Attribute-based key generate.
- DOEncrypt(mpk,(M,π),k)→ct: Policy-based encryption.
- DUDecrypt(ct,sk,S)→k/⊥: Conditional decryption.
- $\mathrm{Revoke}(v_{a}^{\prime },\delta^{\prime })\to Update$: Attribute revocation.
- Trace(sk,uid)→{0,1}: Leakage tracing.

#### 3.2.1. System Initialization: AASetup(λ)→(mpk,msk)

The initial authority AA_0_ generates the system parameters using a security parameter λ:

(1) $$\left(p,G_{1},G_{2},G_{T},e,g_{1},g_{2},H,e{\left(g_{1},g_{2}\right)}^{\alpha }\right)$$

It then selects random numbers $x_{0}\leftarrow Z_{p}$ and $r_{0}\leftarrow Z_{p}$, and computes:

(2) $$\begin{array}{cc} h_{0} & =g_{1}^{x_{0}}, \end{array}$$

(3) $$\begin{array}{cc} h_{0}^{\prime } & =g_{1}^{r_{0}}, \end{array}$$

(4) $$\begin{array}{cc} C_{0} & =C\left(h_{0},h_{0}^{\prime }\right)=g_{1}^{x_{0}}g_{1}^{r_{0}}. \end{array}$$

Subsequently, AA_0_ publishes the initial authority key:

(5) $$AAK_{0}=\left(p,G_{1},G_{2},G_{T},e,g_{1},g_{2},H,e{\left(g_{1},g_{2}\right)}^{\alpha },C_{0}\right).$$

Upon receiving $AAK_{0}$, each of the other authorities AA_i_ performs an identical operation: it selects random numbers $x_{i}\leftarrow Z_{p}$ and $r_{i}\leftarrow Z_{p}$, then publishes its commitment:

(6) $$C_{i}=C\left(h_{i},h_{i}^{\prime }\right)=g_{1}^{x_{i}}g_{1}^{r_{i}}.$$

After *k* authorities have broadcast their commitments, all $C_{i}$ are revealed. The system verifies the validity of each commitment by checking if the equation $C_{i}=C\left(h_{i},h_{i}^{\prime }\right)$ holds. If all checks pass, the protocol proceeds; otherwise, it outputs an error.

Given that all commitments are valid, the authority aggregate public key is computed as:

(7) $$AAPK=\prod_{i=1}^{k}h_{i}.$$

The authorities then collaboratively compute the master key α in a decentralized manner using a Joint Shamir RSS scheme:

- Each authority AA*_k_* generates a random secret $\delta_{k}\in Z_{p}$.
- With randomly chosen coefficients $a_{1},a_{2},\ldots ,a_{t}\in Z_{p}$, each AA*_k_* constructs a polynomial of degree *t* (where *t* is the threshold for reconstructing α):
  (8) $$f_{k}\left(z\right)=\delta_{k}+a_{1}z+a_{2}z^{2}+\ldots +a_{t}z^{t}.$$
- Each AA*_i_* computes and sends the secret share $ss_{i,j}=f_{i}\left(j\right)$ to authority AA*_j_*.
- Each AA*_k_* receives *n* such shares from other authorities and computes its master secret share:
  (9) $$ss_{j}=\sum_{i=1}^{n}ss_{i,j}\;\mathrm{mod}\;q.$$
- Finally, the master key α is reconstructed by any set of *k* authorities using Lagrange interpolation over their shares $ss_{j}$:
  (10) $$\alpha =\sum_{j=1}^{k}ss_{j}L_{j},\;\mathrm{where}\;L_{j}=\prod_{\begin{array}{c} i=1\\ i\neq j \end{array}}^{k}\frac{-i}{j-i}.$$

The revocation value δ is computed similarly through the same protocol.

Let $H:\left[|\theta |+1\right]\to G_{1}$ be a hash function, where θ denotes the set of global attributes. The system’s master secret key and public key are then defined as:

(11) $$\begin{array}{cc} msk & =\alpha , \end{array}$$

(12) $$\begin{array}{cc} mpk & =(p,G_{1},G_{2},G_{T},e,g_{1},g_{2},H,e{\left(g_{1},g_{2}\right)}^{\alpha }). \end{array}$$

#### 3.2.2. Authority-Issued User Keys: AAkeyGen(msk,uid)→(sk)

A user submits their uid to *j* authorities ${\mathrm{AA}}_{j}$ for attribute registration. Each ${\mathrm{AA}}_{j}$ generates a partial secret key for the user:

(13) $${\left\{ssk\right\}}_{j}=g_{1}^{ss_{j}L_{j}}.$$

Let $v_{a}\overset{\$}{\leftarrow }Z_{p}$ be a version number. For a user with an attribute set S⊆θ and each attribute u∈S, a random number $r\overset{\$}{\leftarrow }Z_{p}$ is selected, and the following components are computed:

(14) $$\begin{array}{cc} RK & =g_{1}^{\delta v_{a}}, \end{array}$$

(15) $$\begin{array}{cc} sk_{1} & =\left(\prod_{j\in t}g_{1}^{ss_{j}L_{j}}\right)\cdot H{\left(|\theta |+1\right)}^{r}=g_{1}^{\alpha }\cdot H{\left(|\theta |+1\right)}^{r}, \end{array}$$

(16) $$\begin{array}{cc} sk_{2,u} & =H{\left(u\right)}^{r}\cdot RK^{r}, \end{array}$$

(17) $$\begin{array}{cc} sk_{3} & =g_{2}^{r}, \end{array}$$

(18) $$\begin{array}{cc} sk_{4} & =e\left(g_{1}^{r},g_{2}^{H(uid\|msk)}\right). \end{array}$$

The complete secret key for each attribute u∈S is then constructed as:

(19) $$\begin{array}{c} sk=\left(RK,sk_{1},{\left\{sk_{2,u}\right\}}_{u\in S},sk_{3},sk_{4}\right). \end{array}$$

#### 3.2.3. Data Owner Encryption: $\mathrm{DOEncryption}\left(\mathrm{Enc}\left(Message\right),mpk,\left(M,\pi \right),pp_{u}\right)\to ct$

The Data Owner (DO) constructs an access control structure (M,π) and conceals the original key *k* of Enc(Message) using the system public key mpk and user public key $pp_{u}$.

Let random numbers be generated as follows: $s_{1}\overset{\$}{\leftarrow }Z_{p}$, $v\overset{\$}{\leftarrow }Z_{p}$, $s_{2}\overset{\$}{\leftarrow }Z_{p}$. Given an access structure (M,π) where *M* is an $n_{1}\times n_{2}$ matrix and $i\in [n_{1}]$ denotes a row index, the DO collaboratively generates the ciphertext with authorities ${\mathrm{AA}}_{j}$ corresponding to the attributes in the access policy:

(20) $$\begin{array}{cc} ct_{0} & =k\cdot e{\left(g_{1}^{\alpha },g_{2}\right)}^{s_{1}}, \end{array}$$

(21) $$\begin{array}{cc} ct_{1} & =g_{2}^{s_{1}}, \end{array}$$

(22) $$\begin{array}{cc} ct_{2,j} & =g_{2}^{s_{2}\left[j\right]}, \end{array}$$

(23) $$\begin{array}{cc} ct_{3,i} & =H{\left(|\theta |+1\right)}^{M_{i}{\left(s_{1}\|v\right)}^{⊤}}\cdot H{\left(\pi \left(i\right)\right)}^{s_{2}\left[\rho \left(i\right)\right]}\cdot RK^{s_{2}}. \end{array}$$

The complete ciphertext is constructed as:

(24) $$\begin{array}{c} ct=\left(ct_{0},ct_{1},{\left\{ct_{2,j}\right\}}_{j\in \tau },{\left\{ct_{3,i}\right\}}_{i\in [n_{1}]}\right) \end{array}$$

where:

- In the access control matrix *M*, τ represents the maximum allowable number of repetitions for a single attribute.
- The blinding factor is given by $d=e{\left(g_{1},g_{2}\right)}^{\alpha s_{1}}$.

#### 3.2.4. Data User Decryption: $\mathrm{DUDecrypt}\left((M,\pi ),S,ct,sk\right)\to key$

If the data user’s attribute set *S* is an authorized set under the access control structure (M,π), then there exists a set of constants ${\left\{\gamma_{i}\right\}}_{i\in I}$ such that: $\sum_{i\in I}\gamma_{i}M_{i}=\left(1,0,\ldots ,0\right)$.

Furthermore, since the ${\mathrm{AA}}_{j}$ signatures embedded in the user’s secret key sk coincide with those embedded in the ciphertext ct by the data owner, the following computation can be performed:

(25) $$\begin{array}{cc} key & =e\left(sk_{1},ct_{1}\right)\cdot \frac{e\left(\prod_{i\in I}{\left(sk_{2,\pi (i)}\right)}^{\gamma_{i}},\;ct_{2,j}\right)}{e\left(\prod_{i\in I}{\left(ct_{3,i}\right)}^{\gamma_{i}},\;sk_{3}\right)}, \end{array}$$

(26) $$\begin{array}{cc} k & =\frac{ct_{0}}{key}. \end{array}$$

#### 3.2.5. Attribute Revocation: Revoked→(Update)

The revocation protocol executes the following operations periodically:

The Authority (AA) updates the version number $v_{a}^{\prime }\overset{\$}{\leftarrow }Z_{p}$, collaboratively generates a new revocation value $\delta^{\prime }$.

The AA then publishes Update to non-revoked users and refreshes the following components:

(27) $$\begin{array}{cc} RK^{\prime } & =g_{1}^{v_{a}^{\prime }\delta^{\prime }}, \end{array}$$

(28) $$\begin{array}{cc} \mathrm{Update} & =\frac{RK^{\prime }}{RK}. \end{array}$$

Upon receiving the update, non-revoked users can autonomously update their secret keys:

(29) $$\begin{array}{c} sk_{2,u}^{\prime }=sk_{2,u,j}\cdot \mathrm{Update}. \end{array}$$

Concurrently, the data owner updates the ciphertext using Update:

(30) $$\begin{array}{c} ct_{3,i}^{\prime }=ct_{3,i,j}\cdot \mathrm{Update}. \end{array}$$

#### 3.2.6. Accountability

If a secret key sk is compromised, the Attribute Authority (AA) can initiate a tracing procedure using the component $sk_{4}$.

- Given: User identifier $uid_{n}$ and version parameter $RK_{n}$.
- Compute:
  (31) $$\begin{array}{c} \mathrm{UTK}\leftarrow \frac{e\left(g_{1}^{H(uid\|msk)},\;sk_{3}\right)}{sk_{4}}. \end{array}$$
- Trace: The AA can pinpoint the accountable user by checking if UTK=1.

## 4. Security Proofs

### 4.1. Security Model

We define the security of our revocable attribute-based encryption scheme against chosen-plaintext attacks (R-IND-CPA) via the following security game ${\mathrm{Game}}_{R-\mathrm{ABE}}^{\mathrm{IND}-\mathrm{CPA}}\left(A,\lambda \right)$ between a challenger C and a probabilistic polynomial-time (PPT) adversary A.

Initialization Phase:  The challenger C runs the setup algorithm AASetup(λ)→(mpk,msk), where the master secret key msk is distributed among multiple authorities. C provides the public parameters mpk to the adversary A and initializes the version number $v_{a}$ for each attribute along with a revocation list RL.
Query Phase 1:  The adversary A may adaptively issue a polynomial number of queries to C:
  - Private Key Query $O_{sk}$: A submits an attribute set *S* and a user identity uid. C runs AAkeyGen(msk,uid)→sk and returns the secret key sk to A.
  - Revocation Query $O_{rev}\left(attr\right)$: A specifies an attribute attr. C simulates the attribute authorities to execute the revocation algorithm Revoked→Update, updates the ciphertext to version $v_{a}\overset{\$}{\leftarrow }Z_{p}$, and sends the update information Update to A.
  - Corrupted Authority Query $O_{corrupt}\left(j\right)$: A may corrupt up to t−1 authorities. C returns the internal state (including secret shares) of authority ${\mathrm{AA}}_{j}$ to A.
Challenge Phase:  A submits two equal-length messages $m_{0}$ and $m_{1}$, along with a challenge access policy $\left(M^{*},\pi^{*}\right)$. None of the attribute sets *S* queried in Phase 1 can satisfy $\left(M^{*},\pi^{*}\right)$, and for any revoked attribute attr in $\left(M^{*},\pi^{*}\right)$, A cannot possess a key with version $v_{a}^{new}$ (the latest version after revocation). C randomly selects a bit b←{0,1}, runs $\mathrm{DOEncryption}\left(\mathrm{Enc}\left(m_{b}\right),mpk,\left(M^{*},\pi^{*}\right),pp_{u}\right)\to ct^{*}$, and sends the challenge ciphertext $ct^{*}$ to A.
Query Phase 2:  A may continue to issue a polynomial number of $O_{sk}$, $O_{rev}\left(attr\right)$ and $O_{corrupt}\left(j\right)$: queries as in Phase 1, with the restriction that none of the queried attribute sets *S* satisfy the challenge policy $\left(M^{*},\pi^{*}\right)$. C uses the latest attribute version numbers when generating keys.
Guess Phase:  The adversary A outputs a guess $b^{\prime }$. The advantage of A in this game is defined as:
  (32) $$\begin{array}{c} {\mathrm{Adv}}_{A}^{R\text{-}\mathrm{IND}\text{-}\mathrm{CPA}}\left(\lambda \right)=\left|\mathrm{Pr}\left[b^{\prime }=b\right]-\frac{1}{2}\right|. \end{array}$$

The scheme is said to be secure if for any PPT adversary A, the advantage ${\mathrm{Adv}}_{A}^{R\text{-}\mathrm{IND}\text{-}\mathrm{CPA}}\left(\lambda \right)$ is negligible in the security parameter λ.

Security Properties: The security game ${\mathrm{Game}}_{R\text{-}\mathrm{ABE}}^{\mathrm{IND}\text{-}\mathrm{CPA}}$ captures not only IND-CPA security but also the following properties:

- Collusion Resistance: Even if A obtains multiple private keys from different users and/or corrupts up to t−1 authorities, they cannot decrypt a ciphertext if none of the individual key’s attribute sets satisfies the access policy (M,π).
- Forward Security: A secret key for an attribute at version $v_{a}$ cannot decrypt a ciphertext for the same attribute that has been updated to a newer version $v_{a}^{\prime }$ via a revocation query.
- Backward Security: A ciphertext for an attribute at version $v_{a}$ cannot be decrypted by a secret key for the same attribute that has been updated to a newer version $v_{a}^{\prime }$.

### 4.2. Notations and Encoding Definitions

Following the FABEO scheme, our construction can be defined within the following PES-ABE framework.

System Parameters:

- Master key: $\alpha \in Z_{p}$
- Revocation key: $\delta \in Z_{p}$
- Attribute hash base: $b=(b_{1},\ldots ,b_{\left|\theta \right|},b_{|\theta |+1})$
- Hash function for attributes: $H\left(u\right)=g_{1}^{b_{u}}$
- User identity hash: $t_{uid}=H\left(uid\|msk\right)\in Z_{p}$
- Master secret key: msk=(α,δ)

Secret Key Encoding: For version $v_{a}$ and user attributes u∈S, the secret key is encoded as $sk\left(S,v_{a},uid\right)=\left(k^{1},k^{2}\right)$:

(33) $$\begin{array}{cc} k^{1} & =\left(\alpha +b_{|\theta |+1}\cdot r\;∥\;b_{u}\cdot r+\delta \cdot v_{a,u}\cdot r\right), \end{array}$$

(34) $$\begin{array}{cc} k^{2} & =r. \end{array}$$

Here, $v_{a,u}$ denotes the version number of attribute *u* at key generation. Notably, our scheme introduces an additional verification component $k^{T}=r\cdot t_{uid}.$

Ciphertext Encoding: For an access policy (M,π), the ciphertext is encoded as $ct\left(M,\pi ,v_{a}\right)=\left(c^{1},c^{2}\right)$:
  (35) $$\begin{array}{cc} c^{1} & ={\left\{M_{i}\left(s_{1}\|v\right)\cdot b_{|\theta |+1}+s_{2}\left[\rho \left(i\right)\right]\cdot b_{\pi (i)}+\delta v_{a,\pi (i)}\cdot s_{2}\right\}}_{i\in [l]}, \end{array}$$
  (36) $$\begin{array}{cc} c^{2} & =(s_{1}\|s_{2}). \end{array}$$

Here, $v_{a,\pi (i)}$ denotes the version number of attribute π(i) at encryption time.

Decryption: When the key version matches the ciphertext version and the attribute set *S* satisfies the access policy, the decryption process symbolically recovers $\alpha s_{1}$.

### 4.3. Symbolic Security

If our PES-ABE encoding is symbolically secure, then the system fails to decrypt correctly when either the user’s attributes do not satisfy the access policy, or the user’s attribute version number does not match the current version number.

We prove by contradiction. Specifically, we need to show that for (M,π)∈X and S∈Y, if P((M,π),S)=0, then:

(37) $$\begin{array}{c} \mathrm{span}\left(\tilde{\alpha }⊗c_{x}^{2}\right)\cap \mathrm{span}\left(c_{x}^{1}⊗k_{y}^{2}\|c_{x}^{2}⊗k_{y}^{1}\right)=\left\{0\right\}. \end{array}$$

Here, $x=(M,\pi ,v_{a}^{ct})$ is the label containing the ciphertext version number; $y=(S,v_{a}^{sk},uid)$ is the label containing the key version number; and $\left(\tilde{\alpha },\tilde{\delta },\tilde{b}\right)$ are formal variables.

Assume, for contradiction, that there exists a non-zero vector $e^{*}$ and non-zero coefficient vectors $e_{1}$, $e_{2}$ such that:

(38) $$\left(\tilde{\alpha }⊗c_{x}^{2}\right)\cdot e^{*⊤}=\left(c_{x}^{1}⊗k_{y}^{2}\right)\cdot e_{1}^{⊤}+\left(c_{x}^{2}⊗k_{y}^{1}\right)\cdot e_{2}^{⊤}.$$

If the attribute set *S* does not satisfy the access structure (M,π) yet decryption is possible, there must exist a vector w such that for all π(i)∈S, $\langle w,M_{i}\rangle =0$ and w[1]=1.

Let $({\tilde{s}}_{1}\|\tilde{v})=w$. Then, the polynomial in Equation (38) can be transformed into:

(39) $$\begin{array}{cc} \tilde{\alpha }e_{1}^{*}+\tilde{\alpha }{\tilde{s}}_{2}e_{2}^{*}= & \sum_{i=1}^{l}\left[M_{i}w\cdot {\tilde{b}}_{|\theta |+1}\tilde{r}+{\tilde{s}}_{2}\left[\rho \left(i\right)\right]{\tilde{b}}_{\pi (i)}\tilde{r}+\tilde{\sigma }{\tilde{v}}_{a,\pi (i)}{\tilde{s}}_{2}\tilde{r}\right]e_{1}^{⊤}\\ \; & +\left(\tilde{\alpha }+{\tilde{b}}_{|\theta |+1}\tilde{r}\right){\tilde{s}}_{2}e_{2,1}^{⊤}+\left({\tilde{b}}_{u}\tilde{r}+\tilde{\delta }{\tilde{v}}_{a,u}\tilde{r}\right){\tilde{s}}_{2}e_{2,2}^{⊤}. \end{array}$$

By comparing coefficients:

- For $\tilde{\alpha }$: The term $\tilde{\alpha }e_{1}^{*}$ on the left has no corresponding term on the right. Thus, $e_{1}^{*}=0$.
- For $\tilde{\alpha }{\tilde{s}}_{2}$: The term $\tilde{\alpha }{\tilde{s}}_{2}e_{2}^{*}$ on the left must equal $\tilde{\alpha }{\tilde{s}}_{2}e_{2,1}^{⊤}$ on the right. Hence, $e_{2}^{*}=e_{2,1}^{⊤}$.
- For ${\tilde{s}}_{2}\tilde{r}$: Since $M_{i}w=0$ for π(i)∈S, the equation simplifies to:
  (40) $$\begin{array}{cc} 0= & \sum_{i:\pi (i)\in S}\left[{\tilde{s}}_{2}\left[\rho \left(i\right)\right]{\tilde{b}}_{\pi (i)}+\tilde{\sigma }{\tilde{v}}_{a,\pi (i)}{\tilde{s}}_{2}\right]\tilde{r}e_{1}^{⊤}\\ \; & +\sum_{i:\pi (i)\notin S}\left[M_{i}w\cdot {\tilde{b}}_{|\theta |+1}+{\tilde{s}}_{2}\left[\rho \left(i\right)\right]{\tilde{b}}_{\pi (i)}+\tilde{\sigma }{\tilde{v}}_{a,\pi (i)}{\tilde{s}}_{2}\right]\tilde{r}e_{1}^{⊤}\\ \; & +{\tilde{b}}_{|\theta |+1}{\tilde{s}}_{2}\tilde{r}e_{2,1}^{⊤}\\ \; & +\sum_{u\in S}\left({\tilde{b}}_{u}+\tilde{\delta }{\tilde{v}}_{a,u}\right){\tilde{s}}_{2}\tilde{r}e_{2,2}^{⊤}. \end{array}$$
  This polynomial can be factored as $0=\left(\mathrm{polynomial}\right)\cdot {\tilde{s}}_{2}\tilde{r}$, so all coefficients must be zero. In particular, ${\tilde{b}}_{|\theta |+1}{\tilde{s}}_{2}\tilde{r}e_{2,1}^{⊤}=0$ implies $e_{2,1}^{⊤}=0$, and thus $e_{2}^{*}=e_{2,1}^{⊤}=0$.

This contradicts the assumption that $e^{*}$ is a non-zero vector. Therefore, no such non-zero vectors $e^{*}$, $e_{1}$, $e_{2}$ exist when the attribute set does not satisfy the access policy or the key version mismatches the ciphertext version. Our scheme is symbolically secure under the PES-ABE encoding.

According to the proof in FABEO, if a PES-ABE scheme is symbolically secure, then it also satisfies strong symbolic security. This means the security model can be extended to multiple keys, multiple ciphertexts, and dynamic version queries as defined in ${\mathrm{Game}}_{R\text{-}ABE}^{IND\text{-}CPA}$. Consequently, our scheme also achieves strong symbolic security.

### 4.4. Enhanced Security Analysis

#### 4.4.1. Collusion Resistance Formal Proof

- Multiple Users Collude
  Each user’s secret key contains a unique random value *r*. Consider two users A and B with $r^{\left(A\right)}\neq r^{\left(B\right)}$.
  If they attempt to combine their keys for decryption, they might use components from both users:
  (41) $$\begin{array}{cc} key & =e\left(sk_{1}^{\left(A\right)},ct_{1}\right)\cdot \frac{e\left(\prod_{i\in I}{\left(sk_{2,\pi (i)}^{\left(\mathrm{mix}\right)}\right)}^{\gamma_{i}},ct_{2,j}\right)}{e\left(\prod_{i\in I}ct_{3,i}^{\gamma_{i}},sk_{3}^{\left(X\right)}\right)} \end{array}$$
  (42) $$\begin{array}{cc} \; & =e{\left(g_{1},g_{2}\right)}^{\left(\alpha +b_{|\theta |+1}r^{\left(A\right)}\right)s_{1}} \end{array}$$
  (43) $$\begin{array}{cc} \; & \;\cdot \frac{e{\left(g_{1},g_{2}\right)}^{\left[\sum_{i\in I}\gamma_{i}b_{\pi (i)}r^{\left({\mathrm{src}}_{i}\right)}+\delta v_{a}\sum_{i\in I}\gamma_{i}r^{\left({\mathrm{src}}_{i}\right)}\right]s_{2}}}{e{\left(g_{1},g_{2}\right)}^{\sum_{i\in I}\gamma_{i}\left[M_{i}(s_{1}{\left||v\right)}^{⊤}b_{|\theta |+1}+s_{2}\left[\rho \left(i\right)\right]b_{\pi (i)}+\delta v_{a}s_{2}\right]r^{\left(X\right)}}} \end{array}$$
  where ${\mathrm{src}}_{i}\in \left\{A,B\right\}$ indicates which user’s $sk_{2,\pi (i)}$ component is used for each i∈I, and X∈{A,B} indicates which user’s $sk_{3}$ is used.
  For successful decryption, the $\delta v_{a}$ terms must cancel:
  (44) $$\begin{array}{c} \delta v_{a}\left(\sum_{i\in I}\gamma_{i}r^{\left({\mathrm{src}}_{i}\right)}\right)s_{2}=\delta v_{a}\left(\sum_{i\in I}\gamma_{i}s_{2}\right)r^{\left(X\right)} \end{array}$$
  This requires $\sum_{i\in I}\gamma_{i}r^{\left({\mathrm{src}}_{i}\right)}=\sum_{i\in I}\gamma_{i}r^{\left(X\right)}$, which only holds if all $r^{\left({\mathrm{src}}_{i}\right)}=r^{\left(X\right)}$, that means all components come from the same user. Similarly, the $b_{|\theta |+1}$ terms require $r^{\left(A\right)}=r^{\left(X\right)}$.
  Therefore, colluding users cannot combine partial key components to decrypt a ciphertext that none could decrypt individually.
- Authority Collusion
  Consider the scenario where an adversary compromises up to t−1 attribute authorities, thereby obtaining their secret shares of the master keys.
  With |M|≤t−1 compromised authorities, the adversary obtains shares ${\left\{ss_{j}^{\left(\alpha \right)}=f^{\left(\alpha \right)}\left(j\right)\right\}}_{j\in M}$ and ${\left\{ss_{j}^{\left(\delta \right)}=f^{\left(\delta \right)}\left(j\right)\right\}}_{j\in M}$, where $f^{\left(\alpha \right)}\left(z\right)$ and $f^{\left(\delta \right)}\left(z\right)$ are degree-(t−1) polynomials satisfying $f^{\left(\alpha \right)}\left(0\right)=\alpha$ and $f^{\left(\delta \right)}\left(0\right)=\delta$.
  By the fundamental property of Shamir secret sharing, any set of at most t−1 shares provides *zero information* about the secret. Formally, for any candidate values $\alpha^{\prime },\delta^{\prime }\in Z_{p}$, the conditional probability equals the prior probability:$\mathrm{Pr}\left[\alpha =\alpha^{\prime }|{\left\{ss_{j}^{\left(\alpha \right)}\right\}}_{j\in M}\right]=\mathrm{Pr}\left[\alpha =\alpha^{\prime }\right]$, $\mathrm{Pr}\left[\delta =\delta^{\prime }|{\left\{ss_{j}^{\left(\delta \right)}\right\}}_{j\in M}\right]=\mathrm{Pr}\left[\delta =\delta^{\prime }\right].$
  Consequently, even with t−1 shares, the adversary cannot reconstruct α or δ, compute $g_{1}^{\alpha }$ or $g_{1}^{\delta }$, or generate valid key components $sk_{1}=g_{1}^{\alpha }\cdot H{\left(|\theta |+1\right)}^{r}$ or $RK=g_{1}^{\delta v_{a}}$.
  To demonstrate security rigorously, suppose an adversary A could break IND-CPA security using only t−1 authority shares. We could then construct an algorithm B that takes t−1 shares of an unknown secret *s*, embeds them into a simulation of our scheme, and uses A’s attack to gain information about *s*—contradicting the information-theoretic security of Shamir secret sharing. This reduction argument proves that authority collusion cannot compromise the system’s security.

#### 4.4.2. Forward/Backward Security Proof

The version mechanism ensures:

Forward Security: After revocation to $v_{a}^{\prime }>v_{a}$, new ciphertexts contain $\tilde{\delta }v_{a}^{\prime }{\tilde{s}}_{2}$. Old keys have $\tilde{\delta }v_{a}\tilde{r}$. The mismatch term $\tilde{\delta }\left(v_{a}^{\prime }-v_{a}\right)\tilde{r}{\tilde{s}}_{2}$ prevents decryption.

Backward Security: Symmetrically, new keys with $\tilde{\delta }v_{a}^{\prime }\tilde{r}$ cannot decrypt old ciphertexts with $\tilde{\delta }v_{a}{\tilde{s}}_{2}$.

In our symbolic proof, this corresponds to ${\tilde{v}}_{a,\pi (i)}^{ct}\neq {\tilde{v}}_{a,u}^{sk}$ causing non-cancellation of $\tilde{\delta }$ terms.

### 4.5. Security Reduction

Under the Generic Group Model (GGM), given a security parameter λ, we consider an adversary A that performs at most *Q* group operations and oracle queries in the security game ${\mathrm{Game}}_{R-\mathrm{ABE}}^{\mathrm{IND}-\mathrm{CPA}}\left(A,\lambda \right)$. If our scheme satisfies strong symbolic security, then the advantage of A in ${\mathrm{Game}}_{R\text{-}\mathrm{ABE}}^{\mathrm{IND}\text{-}\mathrm{CPA}}\left(A,\lambda \right)$ is negligible:

(45) $${\mathrm{Adv}}_{A}^{R\text{-}\mathrm{IND}\text{-}\mathrm{CPA}}\left(\lambda \right)\leq \frac{O(Q^{2})}{p}.$$

The security proof proceeds via a sequence of security games.

${\mathrm{Game}}_{0}$: The challenger C and the adversary A interact according to the real scheme in ${\mathrm{Game}}_{R\text{-}\mathrm{ABE}}^{\mathrm{IND}\text{-}\mathrm{CPA}}\left(A,\lambda \right)$. The challenge ciphertext is computed as $ct_{0}^{*}=m_{b}\cdot e{\left(g_{1},g_{2}\right)}^{\alpha s_{1}}$.
${\mathrm{Game}}_{1}$: This game is identical to ${\mathrm{Game}}_{0}$, except that during random oracle queries $O_{H}\left(uid\|msk\right)$, if uid is queried for the first time, the pair $\left(uid,t_{uid}\right)$ is recorded and $t_{uid}$ is returned to A, where $t_{uid}\leftarrow Z_{p}$ is chosen uniformly at random.
${\mathrm{Game}}_{2}$: This game is identical to ${\mathrm{Game}}_{1}$, except that the blinding factor in the challenge ciphertext is replaced. Specifically, $ct_{0}^{*}=m_{b}\cdot T$, where $T=e{\left(g_{1},g_{2}\right)}^{t}$ and $t\leftarrow Z_{p}$ is random. Under the GGM and based on the proven strong symbolic security of our scheme, adversary A cannot distinguish between ${\mathrm{Game}}_{1}$ and ${\mathrm{Game}}_{2}$.

Indistinguishability:

- Transition from ${\mathrm{Game}}_{0}$ to ${\mathrm{Game}}_{1}$: The difference lies in the use of the random oracle model to ensure the randomness of the hash output. The adversary cannot recover msk from the public parameters to distinguish between H(uid‖msk) and the random $t_{uid}$. The advantage loss for A in this transition is ${\mathrm{Adv}}_{A}^{\mathrm{ROM}}\left(\lambda \right)$.
- Transition from ${\mathrm{Game}}_{1}$ to ${\mathrm{Game}}_{2}$: Here, $\alpha s_{1}$ is replaced with a random variable *t*. According to the strong symbolic security, and under the constraints of the security game ${\mathrm{Game}}_{R\text{-}\mathrm{ABE}}^{\mathrm{IND}\text{-}\mathrm{CPA}}\left(A,\lambda \right)$, that here all queried attribute sets do not satisfy the challenge access policy, the polynomial $\alpha s_{1}$ does not lie in the span of the other polynomials. Therefore, A cannot distinguish $\alpha s_{1}$ from a random *t*. By the standard argument of strong symbolic security within the GGM, the adversary’s advantage in this step is bounded by $\frac{O(Q^{2})}{p}$.

In summary, the advantage of the adversary in ${\mathrm{Game}}_{R\text{-}\mathrm{ABE}}^{\mathrm{IND}\text{-}\mathrm{CPA}}\left(A,\lambda \right)$ is:

(46) $${\mathrm{Adv}}_{A}^{{\mathrm{Game}}_{0}}\left(\lambda \right)\leq {\mathrm{Adv}}_{A}^{{\mathrm{Game}}_{1}}\left(\lambda \right)+\frac{O(Q^{2})}{p}\leq {\mathrm{Adv}}_{A}^{\mathrm{ROM}}\left(\lambda \right)+\frac{O(Q^{2})}{p}.$$

This completes the proof.

Therefore, our scheme is IND-CPA secure under the GGM, and also achieves forward security, backward security, and collusion resistance.

## 5. Performance Evaluation

### 5.1. Theoretical Analysis

As shown in Table 1, we compare the key size and ciphertext size of each scheme, and as shown in Table 2, we compare their computational overhead. Our R-T-D-ABE scheme demonstrates significant theoretical advantages across all key performance metrics.

For key and ciphertext size, our scheme achieves a key size of $\left(m+2\right)G_{1}+G_{2}+G_{T}$, which is comparable to the FABEO scheme’s $\left(2m+3\right)G+2G_{T}$. Compared to the structurally simplest OO-MA-CPABE-CRF scheme that does not support revocation, our scheme implements fine-grained attribute revocation by introducing only a minimal number of group elements, achieving an excellent balance between storage efficiency and functional richness. This compactness stems from our design strategy of component embedding rather than module appending. The revocation key RK is integrated into $sk_{2,u}$ ($sk_{2,u}=H{\left(u\right)}^{r}\cdot {RK}^{r}$), avoiding separate storage allocation while adding only one $G_{T}$ element for traceability ($sk_{4}$).

For key generation, the required overhead is ${\left[\left(2m+4\right)E\right]}_{G_{1}}+{\left[1E\right]}_{G_{2}}+P+{\left[mM\right]}_{G_{1}}$, with complexity growing linearly with the number of attributes, *m*. The additional pairing operation (‘P’) primarily stems from the construction of the non-interactive traceability component $sk_{4}=e\left(g_{1}^{r},g_{2}^{H(uid||msk)}\right)$, which introduces only one $G_{T}$ element. Despite supporting multi-authority attribute revocation and accountability—a feature that leads to considerable complexity in MTA-CP-ABE and TR-AP-CPABE—our approach maintains a lower key generation overhead. This efficiency makes it particularly suitable for complex P2P network applications characterized by large attribute sets.

For encryption performance, the required overhead is ${\left[2E\right]}_{G_{2}}+{\left[3lE+2lM\right]}_{G_{1}}$, where complexity scales with the number of rows *l* in the access policy matrix. A key factor contributing to this performance is that our scheme, similar to FABEO, shifts the bulk of the computational load to the smaller $G_{1}$ group. Importantly, the revocation component RK is multiplied as a common factor into each $ct_{3,i}$, adding only constant $G_{1}$ multiplication overhead without changing the asymptotic complexity. This strategic choice results in significantly faster encryption compared to other schemes, highlighting a clear advantage in encryption performance.

For decryption efficiency, our scheme requires only $3P+{\left[2xE+2\left(x-1\right)M\right]}_{G_{1}}+{\left[M+D\right]}_{G_{T}}$ in computational overhead. This is substantially lower than the demanding pairing operations (up to (3|I|+1) or (3x+2)) in other schemes. The minimal 3 pairings result from algebraic cancellation: the RK factors in $sk_{2,u}$ and $ct_{3,i}$ cancel during pairing, while $sk_{4}$ is excluded from normal decryption. This preserves the efficiency of the core CP-ABE structure despite added functionalities. The exceptional decryption efficiency underscores the suitability of our scheme for resource-constrained devices, such as mobile terminals and IoT nodes.

In summary, our scheme demonstrates well-rounded performance across multiple metrics: key/ciphertext size, key generation, encryption, and decryption overhead. It successfully integrates multi-authority attribute revocation and accountability into P2P networks, achieving an effective equilibrium between functionality, efficiency, and security. Consequently, the proposed scheme offers a practical and efficient solution for P2P applications.

### 5.2. Experimental Analysis

We conducted a comparative evaluation of our scheme against several schemes, including FABEO, MTA-CP-ABE, OO-MA-CP-ABE, and R-CP-ABE-Key-Tree. All implementations were executed on an ASUS TUF Gaming A15 laptop equipped with an AMD Ryzen 97940H processor and 16 GB RAM, running Windows 11. The experimental code was developed using the Charm-Crypto library 0.5 and Python 3.7. All tests were conducted under worst-case scenarios, as detailed below:

- For Setup, Key Generation, Encryption, and Decryption Tests: We fixed the number of users to 1, varied the number of attributes from 10 to 500 with a step size of 10, and employed the strictest access policy by connecting all attributes using AND gates only.
- For Ciphertext Update and Key Update Tests: We fixed the number of attributes to 3, set the access policy to (1∧2)∧3, simulated the revocation of attribute 2, and varied the number of users from 10 to 500 with a step size of 10.
- For Accountability Tests: We simulated the worst-case tracing scenario requiring traversal of the entire user list to identify the malicious user, while varying the number of users from 10 to 500 with a step size of 10.

**Figure 2:**
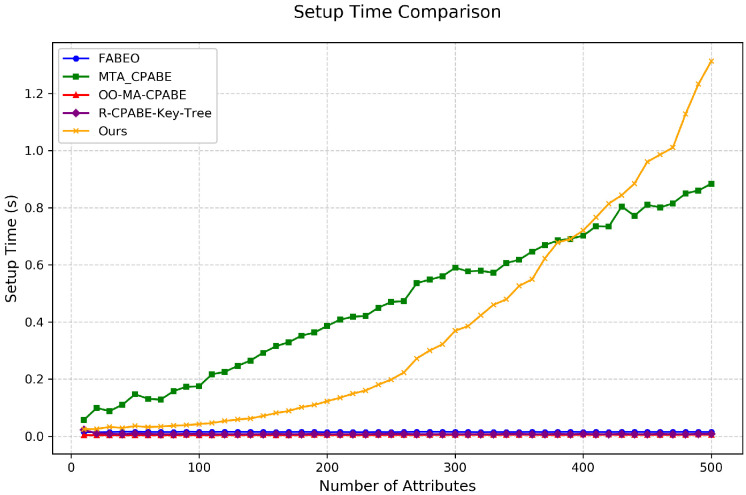
Comparison of setup time among different schemes.

Setup Time: As shown in Figure 2, the setup time of our R-T-D-ABE scheme exhibits a curvilinear growth pattern. Notably, in small-to-medium systems with fewer than 370 attributes, our scheme outperforms MTA-CP-ABE, demonstrating good practicality. Considering that system initialization is an infrequent operation in real-world applications, and our scheme achieves excellent performance in subsequent high-frequency operations such as key generation, encryption, and decryption, this initialization overhead is entirely acceptable. More importantly, our scheme supports both attribute revocation and accountability through a single initialization, providing significant advantages in practical deployment.

**Figure 3:**
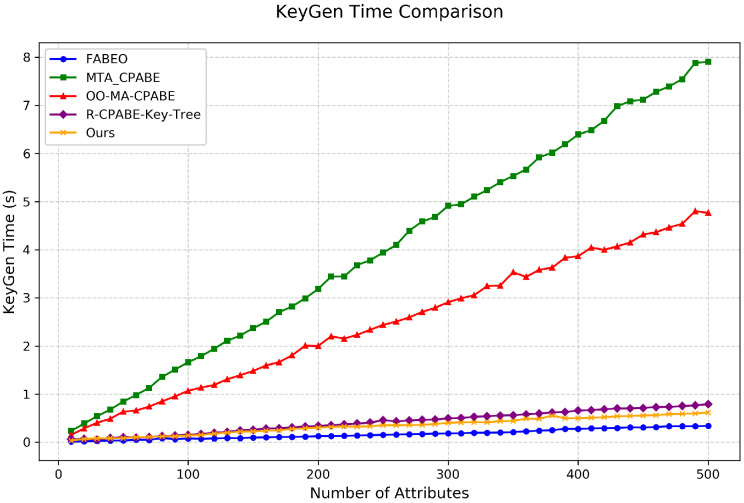
Comparison of key generation time among different schemes.

Key Generation Time: As shown in Figure 3, under the worst-case testing conditions (single user, all-AND policy), our scheme demonstrates exceptional performance in key generation. When the number of attributes reaches 500, R-T-D-ABE requires only approximately 0.6 s to complete key generation, significantly outperforming other schemes, and is only slightly slower than the optimal FABEO scheme (0.3 s). It is noteworthy that our scheme additionally supports revocability and traceability, which are features not offered by FABEO, making the achieved key generation efficiency particularly remarkable.

**Figure 4:**
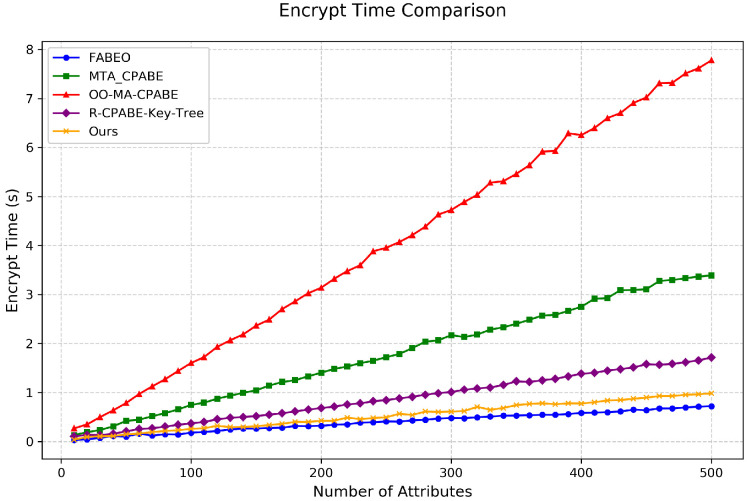
Comparison of encryption time among different schemes.

Encryption Time: As shown in Figure 4, under the worst-case all-AND policy, our scheme maintains encryption performance comparable to the optimal scheme, FABEO. With 500 attributes, our scheme requires 0.9 s for encryption, while FABEO requires 0.7 s. Although slightly slower than FABEO, our scheme still significantly outperforms other comparative schemes. The ability to maintain such high encryption efficiency while simultaneously achieving revocation and traceability functionalities fully demonstrates the superiority of our approach.

**Figure 5:**
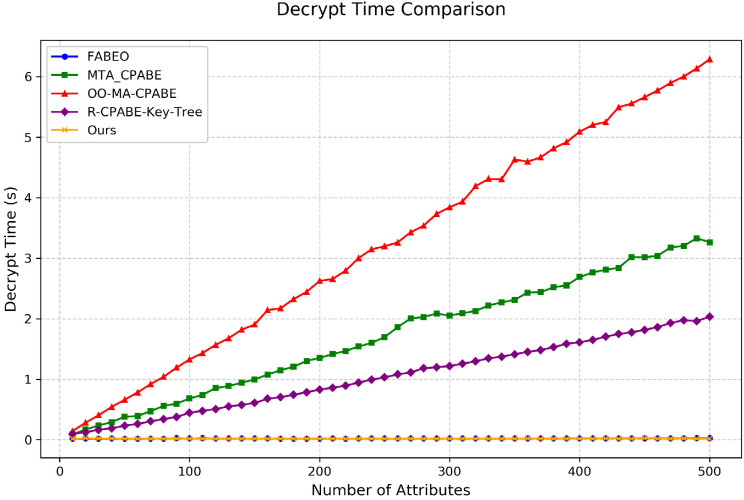
Comparison of decryption time among different schemes.

Decryption Time: As shown in Figure 5, under the worst-case all-AND policy, the decryption performance curve of our scheme almost completely overlaps with that of FABEO, far surpassing other schemes. This excellent performance confirms the high efficiency of our scheme during decryption, making it particularly suitable for P2P networks where nodes often have limited computational resources or require high responsiveness.

**Figure 6:**
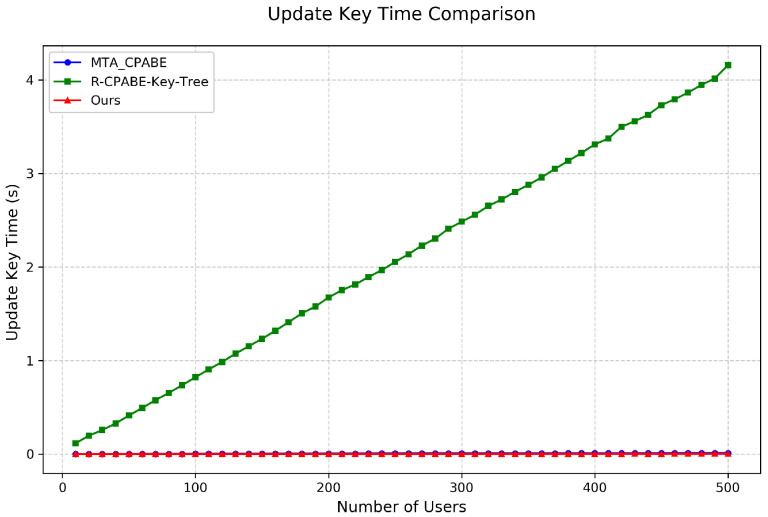
Comparison of key update time among different schemes.

**Figure 7:**
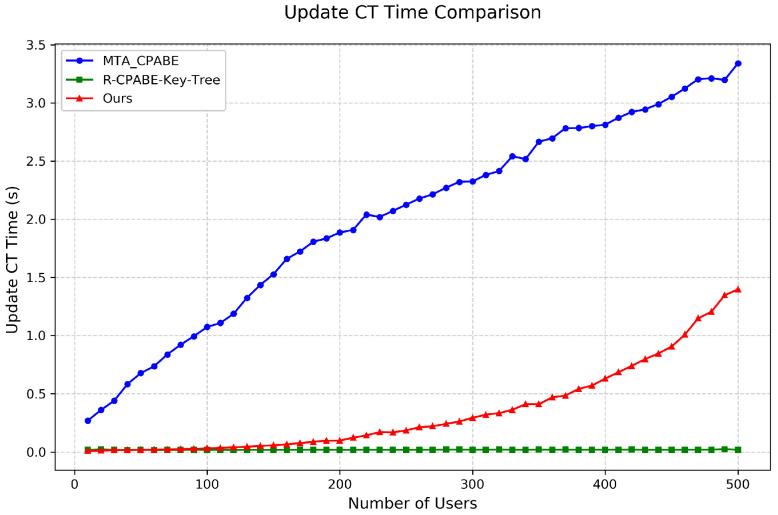
Comparison of ciphertext update time among different schemes.

Key Update and Ciphertext Update Time: As shown in Figure 6 and Figure 7, we tested attribute revocation by fixing the access policy and simulating revocation events. The results indicate:

- Key Update: Our scheme demonstrates outstanding performance in key update. As shown in Figure 6, even with 500 users, the key update time remains below 0.003 s, significantly outperforming R-CP-ABE-Key-Tree. This near-real-time key update capability makes our scheme particularly suitable for highly dynamic P2P network environments.
- Ciphertext Update: As illustrated in Figure 7, our scheme requires only 1.4 s for ciphertext update with 500 users. Although this is slightly higher than the R-CP-ABE-Key-Tree scheme, it is better than MTA-CP-ABE. Notably, the R-CP-ABE-Key-Tree scheme requires up to 4 s for key update. Therefore, considering the overall revocation efficiency, our scheme exhibits a clear advantage.

**Figure 8:**
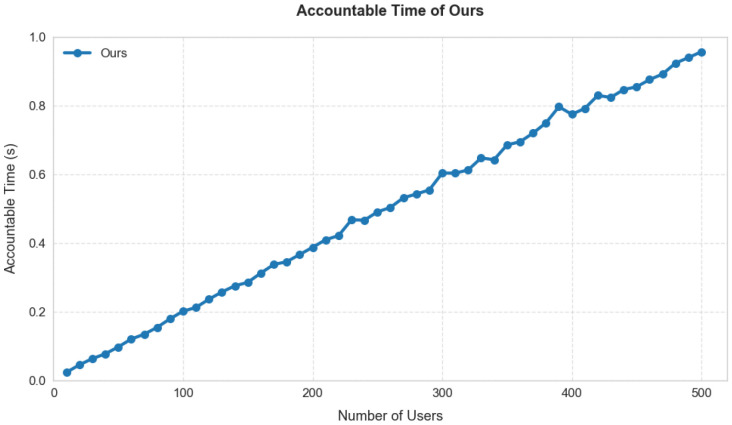
Accountability time of our scheme.

Accountability Time: As shown in Figure 8, we simulated the worst-case accountability scenario (requiring traversal of all users to locate the malicious user). Our scheme requires only 0.95 s for tracing even with 500 users. This result indicates that the traceability feature of our scheme does not introduce significant performance overhead in practical deployment, demonstrating highly efficient tracing capability.

## 6. Conclusions and Future Work

In this paper, we propose a decentralized, revocable, and accountable CP-ABE scheme for P2P networks. By using a threshold-based distributed protocol for master key generation and a user identity binding mechanism, the scheme addresses key challenges in P2P environments: efficiency, centralized trust reliance, and dynamic user management. Theoretical and experimental results show that our scheme retains encryption/decryption performance close to non-revocable schemes while supporting near-real-time key updates and efficient traceability.

Future work will focus on strengthening the formal security proof and extending the scheme to support cross-domain collaboration and dynamic policies in complex P2P scenarios.

## Figures and Tables

**Table 1:** Storage Overhead Comparison.

Scheme	Key Size	Ciphertext Size
FABEO	$\left(2m+3\right)G+2G_{T}$	$\left(3l+1\right)G+G_{T}$
MTA-CPABE	$\left(m+6\right)G+\left(1+m\right)G_{T}$	$\left(4n+3\right)G+G_{T}$
TR-AP-CPABE	$\left(2\log N+m\right)G+lG_{T}$	$\left(n+1\right)G+G_{T}+\log TG$
OO-MA-CPABE-CRF	$\left(m+1\right)G_{1}+G_{2}$	$nG_{1}+2G_{2}$
Ours	$\left(m+2\right)G_{1}+G_{2}+G_{T}$	$lG_{1}+2G_{2}+G_{T}$

**Table 2:** Computational Overhead Comparison.

Scheme	Key Generation	Encryption	Decryption
FABEO	${\left[1M+\left(m+2\right)E+\left(m+1\right)H\right]}_{G_{1}}+{\left[1E\right]}_{G_{2}}$	${\left[lM+2lE+\left(l+1\right)H\right]}_{G_{1}}+{\left[2E\right]}_{G_{2}}$	$3P+{\left[2xE+2\left(x-1\right)M\right]}_{G_{1}}+{\left[M+D\right]}_{G_{T}}$
MTA-CPABE	${\left[\left(d+mK+U+m\right)M+\left(2d+2mK+2U+2\right)E\right]}_{G}$	${\left[\left(2l+1\right)M+\left(3l+t+5\right)E\right]}_{G}$	${\left[\left(3x-1\right)M+xE\right]}_{G_{T}}+\left(3x+2\right)P$
TR-AP-CPABE	${\left[\left(1+m\right)M+\left(2m+5\right)E\right]}_{G}$	${\left[lM+\left(4l+c+2\right)E\right]}_{G}$	${\left[\left(x+1\right)M+1E\right]}_{G}+{\left[\left(3x+2\right)M+\left(x+1\right)E\right]}_{G_{T}}+\left(3x+2\right)P$
OO-MA-CPABE-CRF	(3+2m)E+mM	(2l+1)M+2lE	(3|I|+1)P+3|I|M+1E
Ours	${\left[\left(2m+4\right)E\right]}_{G_{1}}+{\left[1E\right]}_{G_{2}}+P+{\left[mM\right]}_{G_{1}}$	${\left[2E\right]}_{G_{2}}+{\left[3lE+2lM\right]}_{G_{1}}$	$3P+{\left[2xE+2\left(x-1\right)M\right]}_{G_{1}}+{\left[M+D\right]}_{G_{T}}$

**Notations:** *m*: Number of attributes in the attribute set; l,n: Number of rows and columns in the MSP matrix; *x*: Total number of attributes used in decryption; *d*: Height of the user management binary tree; *t*: Bit-length of version number space; *K*: Average number of non-revoked user nodes per attribute; *U*: Total number of non-revoked users; *c*: |cover(R)| (Number of cover nodes for revocation); |I|: Size of attribute set used in decryption; P: Pairing operation; E: Exponentiation operation; M: Multiplication operation; D: Division operation; H: Hash-to-group operation.

## Data Availability

The original contributions presented in this study are included in the article. Further inquiries can be directed to the corresponding author.
